# Modelling early events in *Mycobacterium bovis* infection using a co-culture model of the bovine alveolus

**DOI:** 10.1038/s41598-020-75113-6

**Published:** 2020-10-28

**Authors:** Diane Frances Lee, Graham Roger Stewart, Mark Andrew Chambers

**Affiliations:** 1 School of Veterinary Medicine, University of Surrey, Guildford, Surrey UK; 2 School of Biosciences and Medicine, University of Surrey, Guildford, Surrey UK

**Keywords:** Biological techniques, Immunology, Pathogenesis

## Abstract

Bovine tuberculosis (bTB), a zoonosis mainly caused by *Mycobacterium bovis* has severe socio-economic consequences and impact on animal health. Host–pathogen interactions during *M. bovis* infection are poorly understood, especially early events which are difficult to follow in vivo. This study describes the utilisation of an in vitro co-culture model, comprising immortalised bovine alveolar type II (BATII) epithelial cells and bovine pulmonary arterial endothelial cells (BPAECs). When cultured at air–liquid interface, it was possible to follow the migration of live *M. bovis* Bacille Calmette-Guérin (BCG) and to observe interactions with each cell type, alongside cytokine release. Infection with BCG was shown to exert a detrimental effect primarily upon epithelial cells, with corresponding increases in IL8, TNFα, IL22 and IL17a cytokine release, quantified by ELISA. BCG infection increased expression of CD54, MHC Class I and II molecules in endothelial but not epithelial cells, which exhibited constitutive expression. The effect of peripheral blood mononuclear cell conditioned medium from vaccinated cattle upon apical-basolateral migration of BCG was examined by quantifying recovered BCG from the apical, membrane and basolateral fractions over time. The numbers of recovered BCG in each fraction were unaffected by the presence of PBMC conditioned medium, with no observable differences between vaccinated and naïve animals.

## Introduction

*Mycobacterium bovis*, the predominant causative agent of bovine tuberculosis (bTB), is a member of the *Mycobacterium tuberculosis* complex (MTBC), of which other notable members include *Mycobacterium tuberculosis* and the attenuated *M. bovis* Bacillus Calmette-Guerin (BCG) vaccine strain. Whilst *M. bovis* is primarily considered a pathogen of farmed animals including cattle, buffalo, goats and deer^1^, as well as some sylvatic species, other domestic animal species such as pigs, cats, dogs, horses and sheep are considered spill-over hosts. It is also a zoonosis, considered now by the WHO to be a priority disease to tackle^2^. The Global Tuberculosis Report (2018) provides ‘best estimate’ values of 147,000 cases of human TB attributed to *M. bovis*, resulting in some 12,500 deaths worldwide^3^. However, these figures are thought to be widely inaccurate, due to inability to distinguish between *M. bovis* and *M. tuberculosis* with commonly used tests^4^, resulting in under-diagnosis^5^ and a lack of accurate representation of true *M. bovis* incidence^6^.

Although the infection has been controlled in livestock in most developed countries through implementation of test and slaughter control programmes^7–9^, complete elimination is complicated where there is persistent infection of wildlife reservoirs^10–12^ or where test and slaughter is either logistically or financially unfeasible, or culturally unacceptable^13,14^. In these settings, the development of alternative options for disease control, such as vaccination will require a greater understanding of the host–pathogen interaction, especially early events, would be beneficial.

To-date, studies into mycobacteria-mediated pulmonary pathology have placed significant emphasis on the interactions between mycobacteria and alveolar macrophages (AM). However, alveolar type II (ATII) cells constitute an estimated 15% of the total lung cell population and secrete the four surfactant proteins (SPs) SP-A, SP-B, SP-C and SP-D, which together constitute the first line of defence in the distal lung as surfactant coating the respiratory mucosa^15^. It therefore stands to reason that the interaction of mycobacteria with alveolar epithelial cells (AECs) is important in the initial stages of colonisation of the host following inhalation of *M. bovis*, especially since the downstream immune responses elicited by AECs may contribute to the establishment of infection and pathogenesis through the avoidance of mechanisms such as phagocytosis, apoptosis, and autophagy^16,17^. In particular, the ATII cell plays a major role in host–pathogen interactions^18–21^ and innate immunity^22^, from the initial recognition and amplitude of the inflammatory responses to microbial pathogens via pattern recognition receptors (PRRs), to the secretion of antimicrobial effector molecules such as peptides, enzymes, reactive nitrogen and oxygen species and a multitude of cytokines, chemokines, and growth factors^22–24^.

Although members of the MTBC have been shown to both invade and replicate within ATII cells^25–27^, their role in determining the outcome of mycobacterial infection is largely unknown and under-studied. Current knowledge of early events in the pathogenesis of TB have been gained by use of live animal models and a limited number of in vitro monolayer and co-culture models. For the most part, these have been human focussed^28–34^. As the innate immune response differs between species and/or the strain of mycobacteria under study^35^, models of each key target species would be beneficial. A few in vitro models have been developed with particular emphasis on epithelial and endothelial co-culture at air–liquid interface with the aim of mimicking the alveolar-capillary barrier by close juxtaposition of these two cell types. Such models are reported to more accurately recapitulate the architecture of the distal lung epithelium^28^ and better reflect cell behaviours in the in vivo environment compared to two-dimensional monolayers on plastic surfaces under submerged conditions^28,30^. For example, Birkness et al*.* were able to demonstrate the migration of peripheral blood mononuclear cells from the basolateral to apical chamber of an epithelial/endothelial bilayer culture during *M. tuberculosis* infection^31^, whilst Costa et al*.* recently demonstrated the importance of co-culture, with their finding that the pro-inflammatory response differs from that of a mono-culture (in their case, the human epithelial cell line NCI-H441)^30^.

We recently reported the development of the first co-culture model of the bovine alveolus^36^. This consists of a layer of bovine pulmonary arterial endothelial cells (BPAECs) onto which bovine alveolar type II (BATII) epithelial cells are laid. BATII cells are a novel cell line generated by the lentiviral transduction of particles encoding the catalytic subunit of human telomerase (hTERT) and Simian Virus 40 large T antigen (SV40) onto primary ATII cells from adult bovine lung^36,37^. The epithelial-endothelial co-culture is cultured at air–liquid interface on permeable membranes, to recapitulate rudimentary aspects of the primary bovine alveolus. Here we report the use of this model to follow the apical to basolateral migration of live *M. bovis* BCG and to observe interactions of mycobacteria with each cell type using microscopy and immunohistochemistry techniques, alongside the quantification of IL8, TNFα, IL22 and IL17a release, as key initiators of pulmonary inflammation^38^. Using this model, we could demonstrate internalisation of BCG, migration of BCG across the co-cultured cells and a pro-inflammatory response to BCG infection, in addition to observable epithelial cell damage. Collectively, the resulting data add further evidence to the central role of the ATII cell in pulmonary mycobacterial infection, as well consolidating their position as a specific target for mycobacterial damage.

## Results

### BCG migrates through a co-culture model in a time-dependent manner

**Figure 1:**
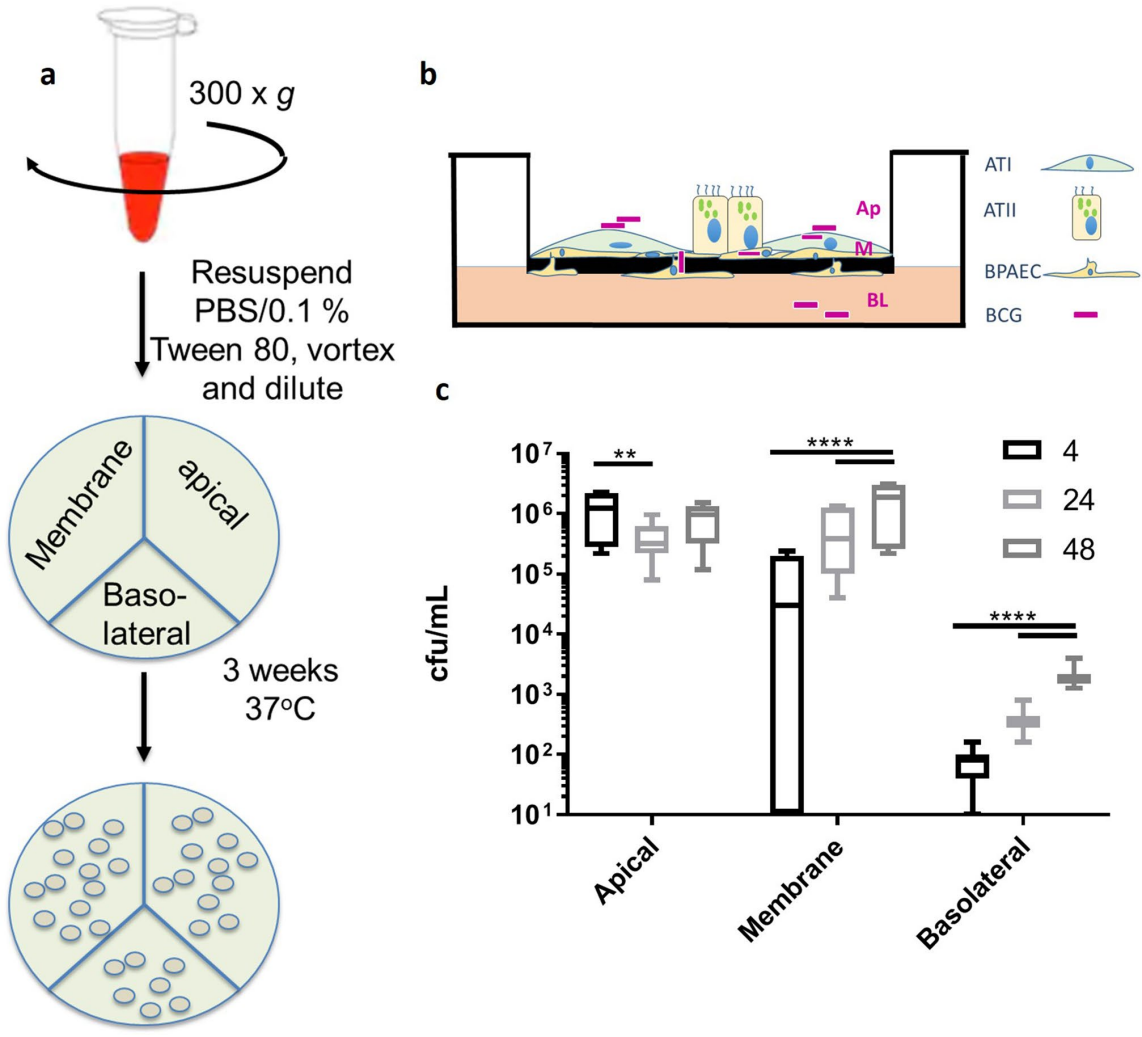
BCG-Pasteur was added to the co-culture model and quantified as outlined in the methods section. (**a**) At 4, 24 and 48 h, supernatant from the apical/basolateral chambers and washed insert membrane was spun at 300 × *g* as described and plated onto tri-partite plates containing 7H11 agar and cultured for 3 weeks at 37 °C. (**b**) The epithelial (BATII) cells differentiated into alveolar type I (ATI) and alveolar type II (ATII) cells when grown at ALI as part of the co-culture model with endothelial BPAECs. Apical (Ap) BCG was defined as those bacilli which could be washed from the apical surface of the co-culture model. Membrane (M) BCG was defined as bacilli isolated from the washed membrane and included BCG released from Triton-X100 lysed epithelial and endothelial cells. Basolateral (BL) BCG was isolated from media taken from the basolateral chamber. (**c**) BCG was quantified by counting colonies and back-calculating to obtain cfu/mL. Both membrane and basolateral fractions demonstrated a time dependent increase in cfu/mL over 48 h.p.i (Two-Way ANOVA; Tukey’s Multiple Comparisons Test; ** P ≤ 0.01, **** P ≤ 0.0001). Data presented as Mean ± SD; n = 12.

The mechanism(s) by which *M. bovis* crosses the alveolar wall to establish infection in the lung is not well understood, therefore any model aiming to represent early invasion of, translocation across, and replication within the ATII cell must demonstrate calculable and replicable migration of mycobacteria. In the current study, BCG was applied apically to co-cultures at an MOI of 10, harvesting and quantifying BCG from the apical, membrane and basolateral fractions at time points of 4, 24 and 48 h, culturing isolated and washed bacteria on 7H11 agar as outlined in the methods (Fig. 1a). Harvested BCG were divided according to region, with apical BCG being defined as on the apical surface of cells (Fig. 1b, ‘Ap’), membrane BCG being defined as contained within epithelial or endothelial cells or the membrane (‘M’) and basolateral BCG being harvested from the basolateral chamber supernatant and wash (‘BL’). BCG was found to decrease significantly in the apical fraction between 4 and 24 h (1.23 × 10^6^ ± 9.63 × 10^5^ to 4.2 × 10^5^ ± 3.07 × 10^5^ cfu/mL) (Two-way ANOVA; Tukey’s Multiple Comparisons Test; P = 0.005), but not between 24 h (4.20 × 10^5^ ± 3.07 × 10^5^ cfu/mL) and 48 h (8.66 × 10^5^ ± 5.52 × 10^5^ cfu/mL; P = 0.184) (Fig. 1c). In the membrane fractions, BCG increased significantly between each time point, ranging from 8.33 × 10^4^ ± 1.02 × 10^5^ cfu/mL at 4 h to 5.87 × 10^5^ ± 5.69 × 10^5^ cfu/mL at 24 h (P < 0.0001) and 1.71 × 10^6^ ± 1.33 × 10^6^ cfu/mL at 48 h (P < 0.001). BCG counts between time points of basolateral samples differed significantly when comparing 4 and 24 h with 48 h, being 76.66 ± 51.76 cfu/mL at 4 h, 3.86 × 10^2^ ± 2.11 × 10^2^ cfu/mL at 24 h (P < 0.0001) and 21.20 ± 9.41 × 10^2^ cfu/mL at 48 h (P > 0.0009 (n = 12, combined from 3 repeats with n = 4 per experiment).

### BCG infection exerts detrimental effects upon barrier integrity and bioelectrical properties

**Figure 2:**
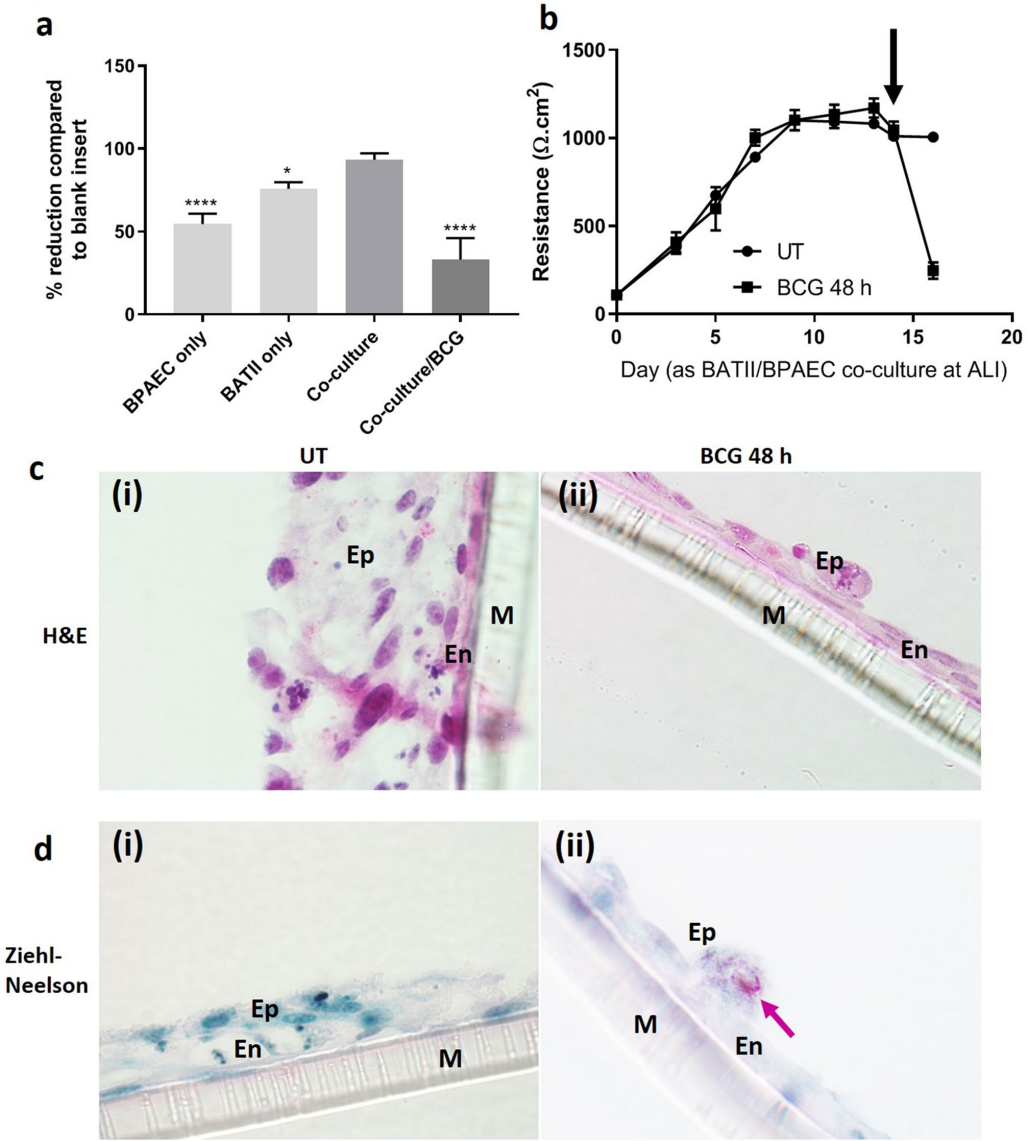
BCG generates catastrophic effects in the epithelial, but not endothelial component of the model. (**a**) Blue Dextran 2000 kDa (DB2000) permeability through endothelial (BPAEC), epithelial (BATII), co-cultures and co-cultures infected with BCG, compared to membrane only. BPAEC monocultures contribute considerably less than BATII cells to the barrier integrity of the co-culture (One-way ANOVA, Tukey’s Multiple Comparisons Test; ****P ≤ 0.0001, *P ≤ 0.05). Barrier properties of the co-culture are significantly reduced upon infection with BCG at MOI 10 (One-way ANOVA; ****P ≤ 0.0001). Data represent 3 experiments performed on different days, presented as Mean ± SD; n = 4. (**b**) TEER, measured from the point at which cultures were raised to air–liquid interface (ALI) until the day of experimentation (day 14; black arrow denotes BCG infection), with a final measurement being performed 48 h.p.i. A significant drop was observed in infected wells, compared to untreated controls (day 16) (Unpaired t-test; P ≤ 0.0001). Data represent 3 experiments performed on different days, presented as Mean ± SD; n = 3. (**c**) Regions of BATII epithelial (Ep) cells in (i) show a healthy and in this instance multi-layered structure, overlaid onto the endothelial (En) cell line BPAEC on the apical side of a permeable membrane (M). H&E staining reveals cuboidal alveolar type II cell morphology, apical microvillae and densely stained regions indicative of lamellar bodies. The underlying endothelial monolayer is distinguished by its elongated and flattened appearance. Following BCG infection (48 h.p.i, (ii)), the epithelial layer is stripped from the underlying endothelial cells almost completely. (**d**) Ziehl–Neelsen (carbol-fuchsin) staining, used in conjunction with the counterstain methylene blue indicates presence of BCG. Methylene blue highlights nuclei as more intensely stained structures, with other cell structures staining less intensely (i). At 48 h.p.i, BCG are observed as multiple red/pink bacilli (pink arrow) in a single surviving epithelial cell (Ep), whilst the methylene blue counterstain reveals a relatively intact endothelial layer (ii). All images taken on the Nikon Eclipse Ci upright microscope using 60 × objective.

Integrity of the co-culture model was determined before, during and after BCG infection, by monitoring the permeability of Blue Dextran in a one-off 60 min measurement as reported previously by Birkness et al.^31^. This was complemented by the determination of trans-epithelial electrical resistance (TEER) using the EVOM2 voltohmeter (World Precision Instruments) fitted with chopstick electrodes. Both Blue Dextran permeability (Blue Dextran 2000 kDa/DB2000) and TEER are considered indicators of tight junction integrity^39,40^, the latter proving particularly useful in the real-time monitoring of tight junction dynamics without cellular damage. Untreated co-cultures were found to impede DB2000 by a mean of 93.27 ± 3.91% over the one-hour treatment period compared to membrane only. This was reduced significantly to 54.55 ± 6.32% across a monoculture of BPAEC cells and 75.86 ± 3.90% across BATII cells only, suggesting that BATII cells played the bigger role in barrier integrity of the model (Fig. 2a). Infection with BCG reduced impedance of DB2000 further to 33.08 ± 12.96%, indicating that the presence of BCG had a detrimental effect upon barrier integrity of the co-culture model (n = 4 for each variable). This was reflected in a drop in TEER values, from a maximum of 1045 ± 54 Ω.cm^2^ on day 14 (at ALI), to 247 ± 47 Ω.cm^2^ on day 16 (48 h post infection (h.p.i)) (Fig. 2b). These data support previous findings which suggest that mycobacteria penetrates the alveolar epithelium by downregulating its bioelectrical properties^41^.

### BCG infection inflicts catastrophic damage to the epithelial component of the model

In addition to the monitoring of barrier integrity by Blue Dextran permeability and TEER measurements, infected co-cultures were fixed, embedded in paraffin wax and sectioned to 0.8 μm, to enable transverse morphological analysis and histochemical staining (hematoxylin and eosin (H&E); Ziehl–Neelsen (ZN)). It was discovered that infection with BCG at a multiplicity of infection (MOI) of 10 over 48 h generated significant damage to the BATII (epithelial) layer of the model, seemingly leaving the BPAEC endothelial layer intact. Characteristic H&E differential staining (Fig. 2c) seen in untreated co-cultures showed cuboidal morphology in the epithelial layers (Ep), vesicular staining reminiscent of lamellar bodies (previously observed in these cells using transmission electron microscopy)^37^ and the darker stained underlying endothelial layer (En) (Fig. 2c(i)). This was reduced to essentially a monolayer of endothelial cells, with a sparse presence of epithelial cells (Fig. 2c(ii)). In staining for acid-fast bacilli (Ziehl–Neelsen), the counterstain of methylene blue showed an intact co-culture of epithelial and endothelial cells (Fig. 2d(i)), whilst BCG were found in high numbers within the sparse number of epithelial cells remaining 48 h after infection (Fig. 2d(ii)).

**Figure 3:**
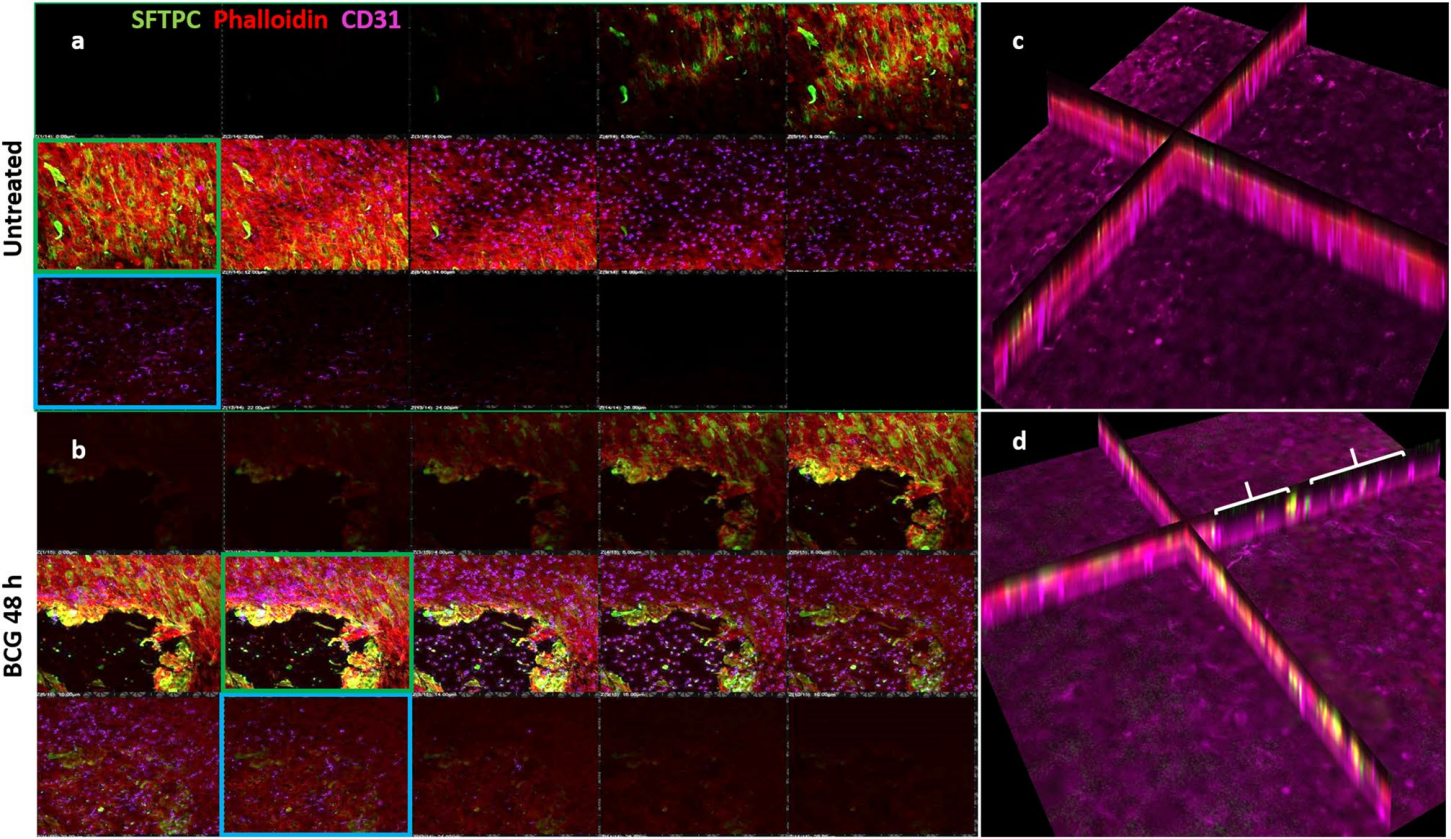
Z-stack confocal images of co-cultures, at 48 h.p.i by BCG, moving from the apical (top left) through to basolateral (bottom right) aspect for each panel. (**a**) Co-cultures of endothelial BPAECs overlaid with the BATII epithelial cell line stained with the ATII marker SFTPC (green), phalloidin (red) and the endothelial marker CD31 (cyan). Moving left to right, a tight epithelial cell layer was observed (red and green fluorescence; green box), giving way to the underlying endothelial BPAEC (red and cyan fluorescence, blue box). (**b**) At 48 h.p.i, the epithelial BATII layer displayed regions of decimation, as evidenced by bare patches in the epithelial layer (red box); however phalloidin staining was still present underneath, in co-localisation with CD31 (blue box), suggesting that endothelial BPAECs were relatively unscathed. (**c**) xz visualisation of the untreated co-culture reflects the integral layers shown in the xy z-stack images of (**a**). (**d**) xz visualisation of the BCG infected co-culture, including regions of catastrophic damage to the epithelial BATII layer (white brackets), whilst CD31 staining is unaffected, suggesting that detrimental effects of BCG infection are limited to the epithelial component of the model. Images acquired using a 20 × objective on a Nikon Ti confocal microscope.

Further examination of the morphology of infected co-cultures was carried out using immunofluorescence and xz confocal microscopy, to enable visualisation of the co-culture in cross-section. Using the ATII marker surfactant protein C (SFTPC), in combination with the endothelial cell marker Cluster of Differentiation 31 (CD31) provided further evidence that co-culture of epithelial and endothelial cells on a permeable membrane resulted in intimate cell–cell contact (Fig. 3). Untreated control cultures contained a heterogeneous population of ATII, identified by SFTPC expression, with differentiated type I cells identified by the absence of SFTPC expression, overlaid onto endothelial cells (Fig. 3a). The integrity of the co-culture was clearly compromised after BCG infection (Fig. 3b), with the detrimental effects appearing to be limited to epithelial cells. This was supported by xz imaging, in which the intact layers can be seen in the untreated cultures (Fig. 3c), with regions of damage (denuding) of the epithelial layer being observed after BCG infection (Fig. 3d).

### Epithelial cell death arises from necrosis rather than apoptosis

**Figure 4:**
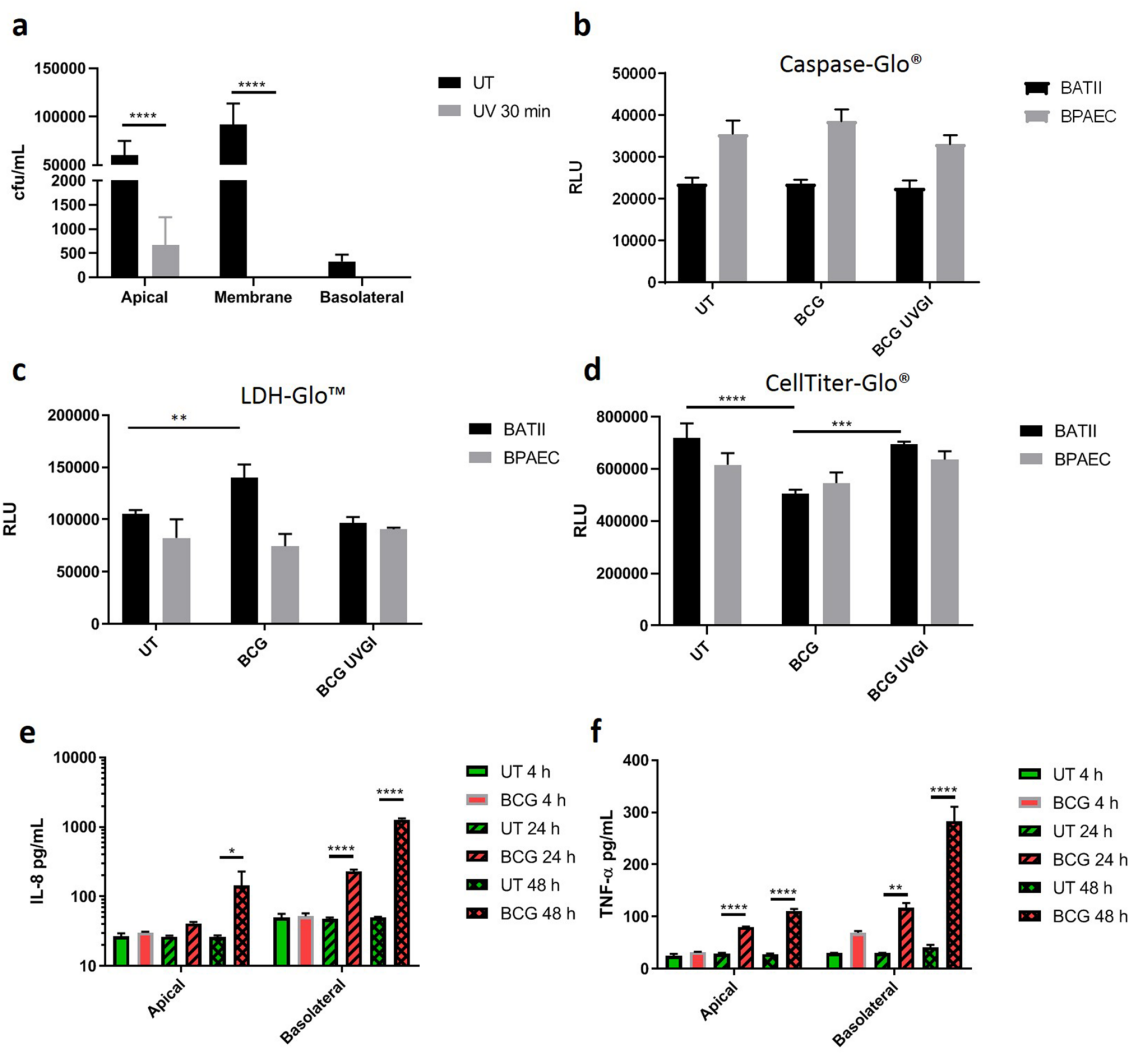
Live BCG interacts with epithelial cells to induce necrosis, but not apoptosis. (**a**) Effect of UV treatment on BCG viability. BCG treated for 30 min under UV light (Ultraviolet germicidal irradiation (UVGI)) demonstrated a significant ‘kill’ efficacy of 99% (Two-Way ANOVA in conjunction with Tukey’s Multiple Comparisons Test; ****P ≤ 0.0001). Data presented as cfu/mL Means ± SD; n = 3. (**b**) No significant increase in caspase 3/7 activity was observed in either cell type when infected with live BCG, compared with untreated (UT) cells over 48 h (Two-Way ANOVA in conjunction with Tukey’s Multiple Comparisons Test; P ≥ 0.05). No effect was detected in cells treated with UVGI BCG. (**c**) LDH cytotoxicity assay revealed significant increase in BATII cell death only with live BCG (Two-Way ANOVA in conjunction with Tukey’s Multiple Comparisons Test; **P ≤ 0.01), but not BPAEC endothelial cell cultures. (**d**) Cell viability as indicated by the CellTiter-Glo assay indicates only live BCG has a detrimental effect on BATII cell viability, with no noticeable effect in BPAEC cultures. Viability was not affected in either culture when infected with UVGI BCG. (**e**) IL8 release both constitutive and in response to BCG is polarised towards the basolateral aspect of the co-culture, showing significant increases at 24 (apical) and 48 h (apical and basolateral) (One-way ANOVA followed by Tukey’s Multiple Comparisons test). (**f**) TNFα release is also polarised towards the basolateral aspect of the culture, showing significant increases at 24 and 48 h in both apical and basolateral fractions. All data for (**e**,**f**) presented as Mean ± SD, n = 3; *P ≤ 0.05, **P ≤ 0.01, ***P ≤ 0.001, ****P ≤ 0.0001.

It was hypothesised that loss of integrity and denuding of the epithelial layer was through cell death, as a direct result of BCG infection. To investigate the mechanisms involved, Caspase-Glo (apoptosis), LDH-Glo (necrosis) and CellTitre-Glo (viability) assays were carried out on BATII epithelial and BPAEC endothelial cells seeded into 96 well formats as mono-cultures and infected either with BCG or UV-irradiated BCG over a period of 48 h. Ultraviolet germicidal irradiation (UVGI) is an established disinfection technique, shown to be effective in mycobacterial species through DNA damage^42–44^. UVGI treated BCG replicates were therefore included to ascertain whether BCG components/ligands were merely required to instigate cell death or if it was an active process brought about during infection by live bacilli (Fig. 4a). It was found that at 48 h.p.i, levels of caspase 3/7 did not alter between groups for BATII or BPAEC cells, regardless of BCG being alive or dead (Fig. 4b). Similarly, no differences were detectable at the earlier time points of 4 and 24 h respectively (Supplementary Figure S1). However, lactate dehydrogenase (LDH) levels of BATII cells, as indicated by the LDH-Glo assay, were significantly increased at 48 h.p.i with live BCG, indicating that cell death was triggered through damage to the plasma membrane of the epithelial cell line^45^ (Fig. 4c). Whilst LDH levels were measured to be higher at 24 h.p.i and 4 h.p.i, these increases were not significant (ANOVA combined with Tukey’s Multiple Comparisons Test; P ≥ 0.05) (Supplementary Figure S2). Contrary to this effect, no significant difference was detected in LDH levels of BPAEC between untreated and live BCG infected cultures at 48 h, whilst no change was observed in LDH levels of either cell type infected with UVGI BCG, suggesting that plasma membrane damage was inflicted actively by live BCG. Viability of BATII monocultures as indicated by the CellTiter-Glo assay was compromised at 48 h.p.i for live BCG only (Two-Way ANOVA, followed by Tukey’s Multiple Comparisons Test; P ≤ 0.0001, n = 3), providing further evidence that cell death arises through a process requiring metabolically active BCG (Fig. 4d; for 4 and 24 h data see Supplementary Figure S3). As with LDH levels, no significant differences were observed for either live or UVGI BCG in BPAEC monocultures.

### IL-8 and TNFα release is polarised in response to BCG infection

The alveolar epithelium has long since been recognised as a source of the chemokine interleukin 8 (IL-8) and cytokine tumour necrosis factor alpha (TNFα) release as part of the orchestrated host response in tuberculosis^46–48^; therefore in the current study, release of these molecules was studied in co-cultures infected with BCG. IL8 was constitutively released at low concentrations both apically and basolaterally. Apical quantities of IL8 were assayed as 26.8 ± 2.7 (4 h), 26.5 ± 1.1 (24 h) and 26.3 ± 1.4 (48 h) pg/mL in untreated co-cultures, whilst basolaterally, IL8 was quantified as 49.8 ± 6.8, 47.6 ± 2.2 and 49.7 ± 1.4 pg/mL at 4, 24 and 48 h respectively (Fig. 4e). Release of IL8 was increased at each time point in response to BCG, particularly into the basolateral compartment. BCG-induced IL8 was determined in the apical compartment to be 30.4 ± 0.8 pg/mL at 4 h, rising significantly to 41.1 ± 1.8 and 143.8 ± 84.1 pg/mL at 24 and 48 h (One-way ANOVA followed by Tukey’s Multiple Comparisons test; P ≤ 0.05, n = 3). This trend was reflected by the release of IL8 into the basolateral compartment of infected cultures, which rose significantly from 52.1 ± 5.3 pg/mL at 4 h, to 229.5 ± 13.4 and 1279.0 ± 52.3 pg/mL at 24 and 48 h respectively (P ≤ 0.0001, n = 3). These findings reflect the role of IL8 as a chemokine and agree with earlier studies of IL8 release^49,50^.

A similar pattern was observed in the release of TNFα (Fig. 4f). Again, concentrations indicative of low-level constitutive expression and release were found in the apical compartment of untreated cultures, ranging from 25.1 ± 3.8 at 4 h, to 29.3 ± 1.5 and 28.1 ± 1.4 pg/mL at 24 and 48 h respectively. Upon BCG infection, no significant rise in TNFα release was seen at 4 h (rising to 32.4 ± 0.5 pg/mL in infected cultures), but significant increases in apical release were observed at 24 h (80.5 ± 0.6 pg/mL; P ≤ 0.01, n = 3) and 48 h (111.1 ± 3.8 pg/mL; P ≤ 0.0001, n = 3). As per IL8, BCG-induced increases were higher in the basolateral compartment, confirming that TNFα release is also polarised in response to BCG infection. Concentrations did not differ considerably from untreated cultures at 4 h (69.1 ± 3.3 pg/mL), but rose significantly at 24 h (117.3 ± 8.7 pg/mL; P ≤ 0.01, n = 3) and 48 h (283.1 ± 27.8 pg/mL; P ≤ 0.0001, n = 3). The polarisation and pattern of TNFα release is indicative of its role as a cytokine that also plays a part in barrier dysfunction, as previously reported^46,51^.

### MHC Class II expression in BATII cells is upregulated in response to BCG infection

**Figure 5:**
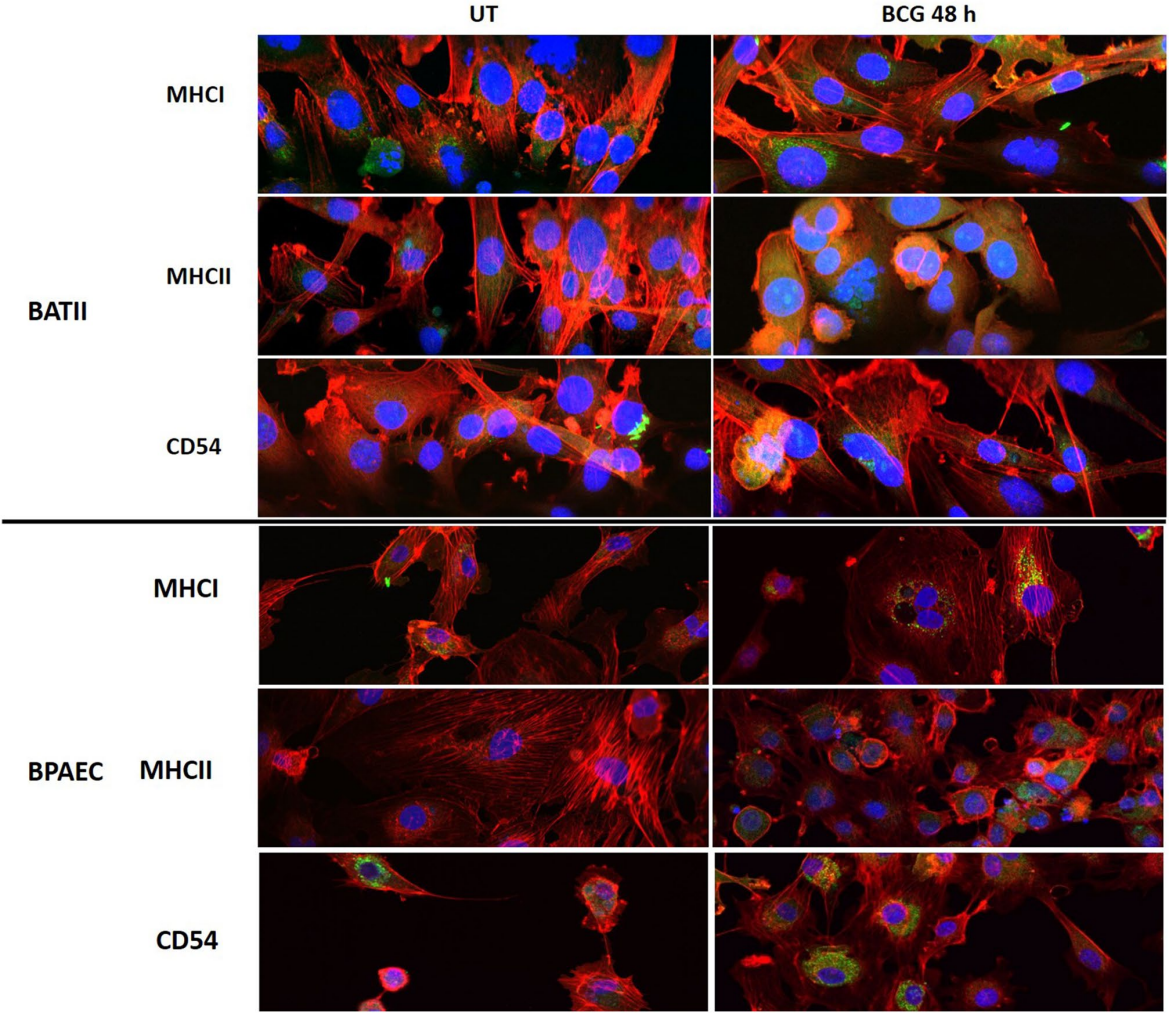
Expression of MHC and co-ordinator molecules in response to BCG infection of BATII and BPAEC cell lines. Cells cultured on coverslips were stained for MHC Class I (MHCI), MHC Class II (MHCII) or CD54 (green; see panel labels), alongside phalloidin (red) and nuclei stained with DAPI (blue). Expression of MHC Class I, MHC Class II and CD54 are constitutive in the untreated (UT) BATII epithelial cell line (upper panel), with a notable rise in expression of MHC Class II and CD54 in response to BCG infection. Whilst MHC Class I and CD54 appear to be constitutively expressed in BPAEC (lower panel) regardless of infection status, MHC Class II is negligible/absent from UT cells. All images taken with 60 × objective.

ATII cells have previously been shown to act as antigen presenting cells, utilising the MHC Class II pathway and upregulating MHC Class II and the co-stimulatory molecule CD54 in response to mycobacterial infection in mice and humans^52^. We therefore examined the expression of both MHC Class I and II along with CD54, in the BATII cell line in the presence or absence of BCG, at 24 h.p.i, comparing expression with that found in the BPAEC endothelial cell line (Fig. 5). Constitutive expression of MHC Class I was observed in the BATII epithelial cells, localised to the perinuclear region. MHC Class II expression appeared more diffuse, with a notable increase in signal from infected BATII cells. Similarly, an increase in CD54 was observed, consistent with previous findings that expression of both CD54 and MHC Class II are increased upon mycobacterial infection^52,53^. By contrast, whilst BCG infected BPAECs expressed MHC Class I, II and CD54 in abundance, the expression of MHC Class II did not appear constitutive and was not observed in uninfected cells, consistent with previous findings that endothelial cells lose MHC Class II expression in vitro^54^.

### IL17a and IL22 response to BCG infection in PBMC conditioned medium differs according to donor vaccination history, with no observed effect upon BCG migration

Previous investigations into the relationship between cytokine and chemokine expression in the context of BCG vaccination have highlighted the role of IL17 and IL22 as biomarkers of a vaccine protected phenotype^55,56^. Additionally, a recent study into the impact of donor vaccination history upon early events in *M. tuberculosis* infection demonstrated a lower level of expression for multiple cytokines, including IL17, and decreased mycobacterial load in PBMCs from BCG vaccinated donors^57^. During the current study, in a researcher blinded experiment, this relationship was examined using PBMCs from cattle exhibiting a strong vaccine protected phenotype (VPP) (PBMC sample 1412) and compared with PBMCs from naïve cattle (PBMC sample 1406).

**Figure 6:**
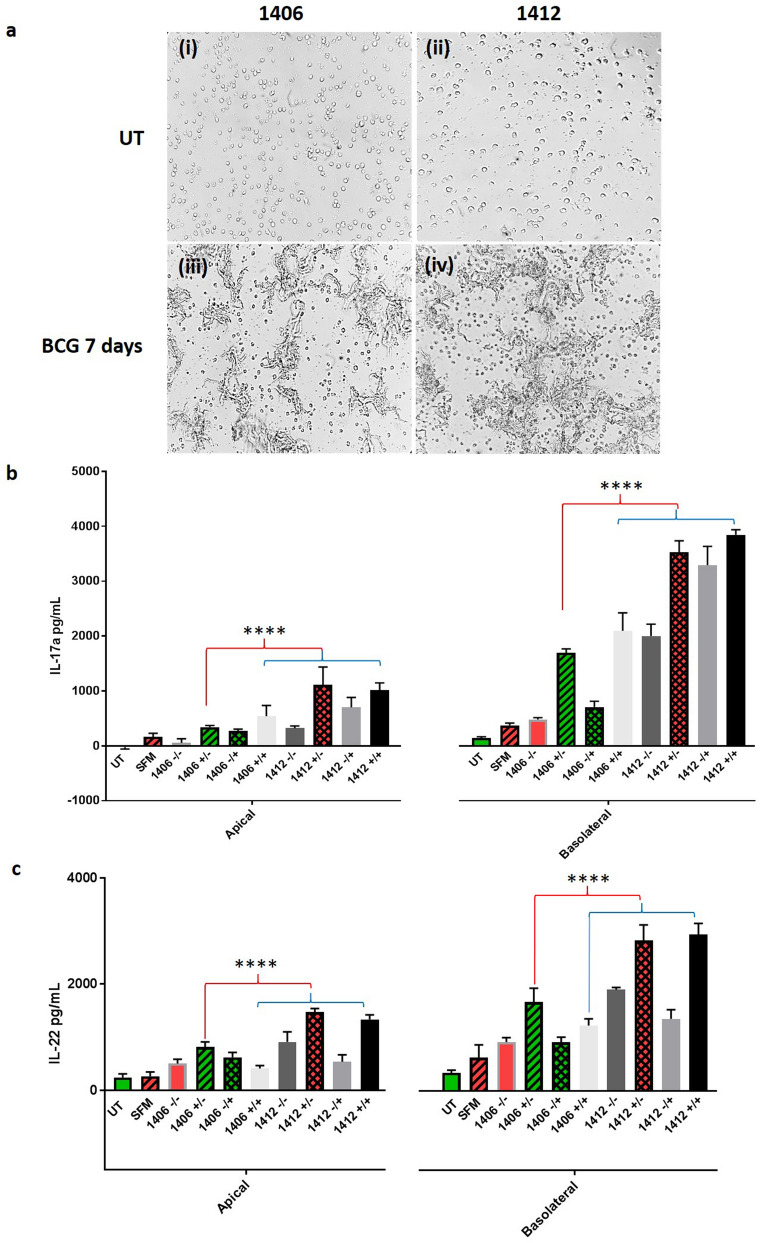
PBMCs from vaccinated/protected or naïve cows were infected with BCG for 7 days, in a blind format. (**a**) Two donated vials of PBMCs identified only as ‘1406′ and ‘1412’ were infected over a 7 day period with BCG (MOI = 1). Untreated cultures of PBMCs 1406 (i) and 1412 (ii) exhibited normal PBMC morphology. BCG infected cultures exhibited multi-nucleated giant cell (syncytial) formation in both 1406 (iii) and 1412 (iv). Images representative of n = 4, objective 20 ×. (**b**,**c**) Following 72 h treatment (48 h.p.i) with CM or controls (UT, untreated (in EGM-2); SFM, OpTmizer CTS serum free medium), apical and basolateral medium harvested from ALI co-cultures was subjected to ELISA for quantification of IL17a and IL22 respectively and analysed using Two-way ANOVA followed by Tukey’s Multiple Comparisons test. Data presented as Mean ± SD; n = 3 (****, P ≤ 0.0001).

When PBMCs were infected with BCG over 7 days, what appeared to be multi-nucleated giant cell (MGC) formed (Fig. 6a). This was particularly evident in the culture later identified as a strong VPP (1412; Fig. 6aii/iv). Filtered (0.2 μm) supernatant (hereon referred to as conditioned medium, CM) harvested from untreated (-) or BCG (MOI of 1) infected ( +) cultures was used to treat ALI BATII/BPAEC co-cultures basolaterally, before the addition of BCG at MOI 10/EGM (100 μL) apically (summarised in Table 1).

**Table 1:** Summary of groups according to infection status of PBMC used to generate conditioned medium (CM) and infection status of co-cultures following a 24 h incubation with CM (total contact time of CM 72 h).

Annotation in text	PBMC infection status (7 d submerged culture)	Co-culture infection status (48 h, ALI)
−/−	−	−
−/+	−	+
+/+	+	+
+/−	+	−

After 48 h, medium was harvested from the apical and basolateral chambers of treated and control co-cultures, for quantification of IL17a (Fig. 6b) and IL22 (Fig. 6c) by enzyme-linked immunosorbent assay (ELISA).

In both apical and basolateral samples, significantly higher quantities of IL17a and IL22 were present in CM generated from PBMCs taken from an animal exhibiting a strong VPP (1412) (Fig. 6b,c respectively; Two way ANOVA followed by Tukey’s Multiple Comparisons Test; P ≤ 0.0001). The lack of significance in cytokine quantity between CM from PBMC cultures infected with BCG (+/−) and samples from co-cultures that were also infected with BCG (+/+) indicates that the vast majority of IL17a and IL22 secretion in response to BCG infection occurred in the initial PBMC culture, a finding consistent with previous reports that these cytokines are predominantly secreted by adaptive (CD4^+^ T cells) or innate lymphocytes (γδ^+^ T cells, NK cells, innate lymphoid cells)^58^. In line with this, the findings of the current study are that the vast majority of cytokine in either case is to be found in the basolateral compartment of co-cultures, to which CM was added.

Significantly higher quantities of IL17a were found in CM originating from naïve (1406) PBMCs only when BCG infection was carried out in PBMC culture (+/− and + / +); Two-way ANOVA followed by Tukey’s Multiple Comparisons Test; P ≤ 0.0001), when compared to OpTmizer SFM alone. Conversely, all CM derived from strong VPP PBMCs (1412) was found to contain significantly higher quantities of IL17a, regardless of BCG infection status of either the PBMC 7-day culture or co-culture (P ≤ 0.0001). These discoveries were replicated in the IL22 data, with the exception that CM+/+ (infections performed in PBMC *and* co-cultures) contained an insignificantly greater quantity of IL22.

**Figure 7:**
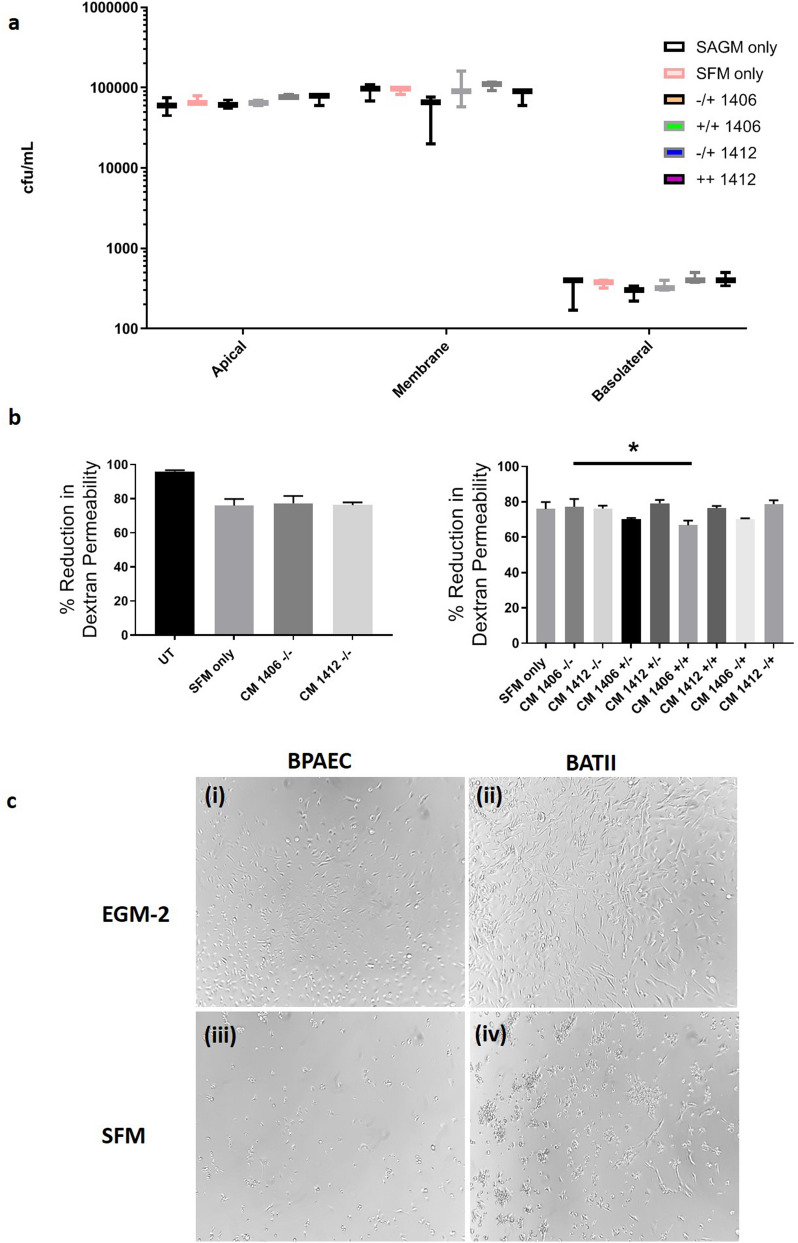
No change in migration of BCG in presence of strong VPP or naïve CM. (**a**) No differences were observed when comparing the migration of BCG through co-cultures treated with CM from naïve and strong VPP PBMCs (One way ANOVA; p ≥ 0.05). Data presented as Mean ± SD, n = 3. (**b**) Dextran permeability studies highlighted that OpTmizer CTS serum free medium (SFM) had a detrimental effect on the integrity of the co-culture, as indicated by an increase in DB2000 quantified in the basolateral chamber. Comparing groups to SFM as negative control, only one point of significance was determined, between the naïve (1406) −/− and + / + groups (One way ANOVA followed by Tukey’s Multiple Comparisons test; *P ≤ 0.05) Data presented as Mean ± SD, n = 3. (**c**) BPAEC (i) and BATII (ii) were cultured in EGM-2 alongside OpTmizer CTS serum free medium (SFM) (iii and iv respectively) for 48 h (the period of exposure of co-cultures to conditioned medium) on LabTek II chamberslides. A decrease in cells was observed for both BPAEC and BATII cultures in SFM, based upon morphological light microscopy analysis. Images representative of n = 4, objective 10 x.

No changes in BCG apical to basolateral migration rate could be determined (Fig. 7a). However, it was noted that a significant increase in DB2000 permeability occurred when co-cultures were treated with OpTmizer SFM only (Fig. 7b). Combined with the observations of a slight increase in IL17a/IL22 and a visible deterioration in cell morphology (Fig. 7c) in co-cultures treated with OpTmizer SFM only, we suggest that there may be a detrimental effect on co-culture integrity of this medium.

## Discussion

The current study aimed to investigate the co-culture system of BATII epithelial cells overlaid onto BPAEC endothelial cells as a viable means to study bovine respiratory infections, including but not exclusively bovine tuberculosis (bTB), and to test the co-culture system as a model by which host–pathogen interactions, vaccinology and toxicology studies may be performed. Two main objectives were thus formulated. The first was to identify and characterise the model in terms of response to infection, beginning with the live attenuated vaccine Bacillus Calmette-Guérin (BCG) as a surrogate to the pathogenic *M. bovis*, to include cytokine response, cell morphology, integrity of the co-culture and apical to basolateral migration of the mycobacteria. The second was to demonstrate the functional utility of the model by testing the hypothesis that a significant aspect of vaccine-mediated protection against bTB is expressed at the level of host–pathogen interactions within the alveolus, by introducing PBMC conditioned medium derived from animals with a naïve (not vaccinated) or strong vaccination protected phenotype (VPP).

Initial studies utilised the co-culture model grown at air–liquid interface, as previously published by the authors^36,37^. By rigorous washing of the membrane and recovery by centrifugation of BCG from the apical and basolateral compartments, the time-dependent tracking of migrating BCG was determined to be possible over a period of 48 h.p.i. The current study did not extend this period, predominantly since the integrity of the co-culture was adversely affected after 48 h, as indicated by increased Blue Dextran 2000 kDa (DB2000) transport and trans-epithelial electrical resistance (TEER), which would affect the sensitivity of the data. It is possible that reducing MOI would have lessened the adverse effects observed here but would not have allowed us to detect differences in the number of BCG migrating across the model. We chose an MOI for the current study equivalent to that used by Birkness et al. for studying the migration of *M. tuberculosis*^31^. Interestingly, they also reported specific damage to the epithelial layer, as opposed to the endothelial component of their bilayer model, suggesting the difference in susceptibility between epithelial and endothelial cells to virulent mycobacteria is real. To further investigate deterioration of the co-culture model in response to BCG infection, we performed immunohistochemistry and Ziehl–Neelsen staining. This provided valuable insight into the adverse effect of BCG infection, biased towards epithelial cells. Whilst the endothelial BPAEC component of the model escaped relatively unscathed, the epithelial layer was all but stripped from the co-culture (Figs. 2 and 3), with multiple bacilli found within a single epithelial cell, in agreement with previous studies^31^. Although it was difficult to measure the percentage of cells infected because they detached as a result of infection. We estimate that 30–40% of epithelial cells contained 10–100 internalised bacteria. In our previous characterisation of the model, epithelial cells were determined to be the main contributor to TEER, a finding replicated in the current study. It was to be expected, therefore, that damage to the epithelial layer would give rise to a substantial drop in both TEER and resistance to DB2000 permeability; the latter also observed by Birkness et al.^31^. These marked declines in TEER and equivalent short-circuit current of the epithelial layer indicate a reduction in the capacity to maintain tight intercellular junctions. TEER is a measure of the permeability of the cell layer to ionic species^40^ and is often used to indicate barrier integrity. It is so far unclear as to why the epithelial layer should be so dramatically affected in this way over endothelial cells. Other authors have reported similar findings: the addition of Influenza A virus (IAV) to a human version of our co-culture model resulted in significant reduction to epithelial-endothelial barrier integrity, primarily through disruption of tight junctions between the epithelial cells^59^. In the case of mycobacteria at least, TNFα appears to cause a reduction in barrier integrity, speculated to be the result of its effect on tight junctions^41,60^. Interestingly, TNFα did not appear to mediate this effect with IAV infection^59^. In the present study, TNFα secretion was elevated in response to BCG infection (Fig. 4), as was the chemokine IL8, both most notably from the basolateral aspect, although it is unclear at this stage whether this preceded the deterioration of the epithelial layer. Lung epithelial cells are a known source of IL8, with its polarised secretion being attributed to its role as a recruiter of neutrophils and T cells^47,50^. Other authors have proposed that cytotoxicity arising from *M. tuberculosis* infection occurs through necrosis and that pro-inflammatory cytokines disrupt the epithelial barrier independently of apoptosis^61,62^. The current study tested this hypothesis by running a series of assays on 2D monocultures of either BATII or BPAEC cells infected over 48 h. These revealed no significant increase in caspase 3/7 levels, an indicator of apoptosis; however lactate dehydrogenase (LDH) levels rose significantly, thus our findings are in agreement with those of Dobos et al*.*^61^ The lack of effect on caspase 3/7, together with the observed BCG-induced increase in TNFα, suggest a TNFα-induced caspase independent necroptosis mechanism^63^, which warrants further investigation. This effect was not reproducible in experiments performed using ultraviolet germicidal irradiation (UVGI) treated BCG, indicating that live bacilli are required to cause cell damage. Interestingly, the same study reports some strains, including *M. bovis* BCG, do not have this adverse effect on epithelial cells (in this case A549)^61^. It is important to note, though, that cell lines will almost certainly exhibit subtle differences in genotype and phenotype. The same authors report that A549 cells do not secrete biologically active TNFα, in contrast to other models^64,65^, thus caution should be exercised when comparing data between cell lines. The A549 cell line has limited ability to form a tight layer (TEER values of 45 Ω.cm^2^) ^40^, reducing its value for studying changes to epithelial barrier integrity.

To address the second objective of demonstrating the functional utility of the co-culture model, the current study tested the hypothesis that vaccine-mediated protection from bTB was centred upon host–pathogen interactions at the epithelial surface. Concerns with regards to tissue incompatibility arising from the use of PBMCs from other animals rather than the donor, from which BATII were generated, led to the use of conditioned medium (CM) in this instance, with the aim of moving onto PBMC cultures for future studies. Donor lung tissue was sourced from a local abattoir facility, where PBMC harvest was not possible; a recognised limitation of the current model. For this reason, PBMCs obtained during previous studies at the Animal and Plant Health Agency (APHA, Weybridge, UK) were used to test the hypothesis that CM from strong VPP PBMCs would restrict better the migration of mycobacteria through the epithelium to endothelium (apical to basolateral). The preparation of CM was performed using PBMC cultures on 2D plastic to maximise supernatant recovery post infection. At 7 days, many aggregations were observed, which the authors propose to be multi-nucleated giant cells (MGCs), in line with previous observations^57,66,67^. MGCs are known to arise from the fusion of multiple macrophages in response to mycobacterial infection in cattle^68–70^. Whilst the number of these cells was not quantified, there appeared to be a higher density in PBMCs from an animal with a strong VPP; a so far unreported observation (search performed May 2020). The authors propose that this merits further study, particularly as this experiment utilised PBMCs from only one animal of each phenotype. Despite these observations, no change in the rate of apical-basolateral migration of BCG could be found in the current study; however adverse effects were observed in air–liquid interface co-cultures of epithelial and endothelial cells treated with the chosen serum free medium, OpTmizer CTS, which most likely affected this result. These included a rise in the permeability of DB2000, an indication of loss of integrity similar to that observed following BCG infection. Culture of both cell types on a 2D surface with OpTmizer resulted in visibly deteriorated cells, for reasons that are not clear. This medium was chosen for its proven record in the culture of T cells^71^, in order to maintain a serum free environment and limit any serum induced effects on cell phenotype. The use of animal serum in cell culture has the drawback of batch variance and has been shown to affect gene transcription and phenotype^72^, thus the authors maintain that serum free culture is important to this type of study, to enable accurate reproducibility by other groups. Future work related to this study will therefore involve further investigations with regards to media formulation.

Whilst cytokines IL17 and IL22, primarily secreted by cells of the adaptive and innate immune system work synergistically to induce lung inflammation^73^, very few IL17 producing cells are present in healthy lungs. Additionally, IL22 acts as a double agent during respiratory infection and contributing to the pro-inflammatory response of IL17a in allergic asthma^74^. In the absence of IL17a, IL22 appears to protect against pathology^75^ and in some cases promote proliferation of epithelial cells^76^. The relationship of these two cytokines in the context of tuberculosis is therefore of considerable interest. It has been proposed that altering the ratio of these two cytokines by regulating IL17a independent of IL22 may influence the outcome of respiratory pathogen invasion^58^. The current study examined these two cytokines in CM generated from PBMCs of a naïve (unvaccinated) and strong VPP phenotype. A marked increase in ELISA quantified IL17a from PBMCs was revealed in uninfected samples from the strong VPP animal when compared with naïve PBMCs, in agreement with earlier findings^57^; however findings from these experiments contradict in terms of IL17a response to further infection with BCG. The earlier study saw no significant difference in IL17a response when a further mycobacterial infection was performed, whilst the current study showed a significant further increase, mimicked by a corresponding increase in IL22. Important to note are the differing strains used when considering such diverse results. In the earlier study, the *M. tuberculosis* H37Ra was used, a strain considered avirulent compared to its close relative H37Rv, with 74 cytoplasmic and 89 integral membrane protein variations between these two strains alone^77^. These differences strongly suggest a role in discrepancies observed between these two strains^78^ and it is not unreasonable to assume that similar discrepancies would occur between *M. tuberculosis* H37Ra and *M. bovis* BCG. Consequently, caution needs to be exercised when comparing studies.

Other researchers report that the immune responses of PBMCs differ with regards to adenosine deaminase and IFNγ activity according to the number of doses of BCG given^79^. Whilst the current study did not examine these particular components of the immune system, infection of the co-culture model in the presence of CM from PBMCs already primed by BCG showed no significant further increase in either IL17a or IL22. This indicated two factors, that IL17a and IL22 are expressed at very low levels by the respiratory epithelium and that any beneficial effect of repeat BCG dosage is independent of these two synergising cytokines.

The current study was inconclusive with regards to the hypothesis that a significant aspect of vaccine protect arises as a result of host–pathogen interaction in the alveolus, for reasons already discussed. However, the BATII/BPAEC co-culture model offers the potential to explore mechanisms of *M. bovis* host–pathogen interaction and to elucidate the mechanisms by which alveolar type II epithelial cells contribute to innate immunity. In a similar vein, the model provides a platform to explore the means by which epithelial cells contribute to, or may be the target of, mycobacteria-mediated pulmonary pathology, an area underrepresented in the literature. Sensitivity to the SFM chosen indicates a drawback to the cell line used; nevertheless, the data presented show the potential of the model for future in vitro studies of host–pathogen interaction and translocation across, and integrity of, the alveolar-capillary barrier more generally.

## Methods

### Mammalian cell culture and assembly of co-culture model

Bovine Pulmonary Arterial Endothelial Cells (BPAECs) (European Collection of Authenticated Cell Cultures, Salisbury, UK) were revived from liquid nitrogen (passages 4–10 were used in the current study) and cultured to 80% confluence in endothelial growth medium 2 (EGM-2) (PromoCell GmbH, Heidelberg, Germany). Cells were trypsinised using 0.25% trypsin/0.05% EDTA and neutralised using an equivalent volume of DMEM/10% FBS, before seeding onto the apical surface of 6.5 mm diameter (0.33 cm^2^) Transwell-CLEAR 8 μm pore size permeable membranes (Corning Inc, Corning, NY) in a 24-well plate, at a density of 2 × 10^5^ cells/insert, (approximately 6.5 × 10^5^ cells/cm^2^). EGM-2 (600 μL) was added to the basolateral chamber of each well. BPAECs were cultured for 5–7 days, replacing EGM-2 in the basolateral chamber and removing apical medium which had seeped through from the basolateral side of the membrane. Bovine Alveolar Type II (BATII) cells were derived from primary ATII cells isolated and immortalised as described previously^36,80^. For the current study, passages 14–18 were used. Cultures were fed every 2–3 days with small airway growth medium (SAGM, Lonza AG, Basel, Switzerland) and passaged accordingly at 70–80% confluence. BATII cells were revived 3 days after BPAEC and cultured in 2D, feeding every 2–3 days in SAGM (SAGM, Lonza AG, Basel, Switzerland) without antibiotics. On the day of seeding, cells were trypsinised, resuspended in EGM-2 and counted, before seeding on top of the BPAEC layer at a density of 2 × 10^5^ cells/insert, as before. Co-cultures were returned to the incubator for 2 h to allow for attachment, after which the apical medium was removed, and the cells cultured at air–liquid interface for 14 days, feeding every 2–3 days basolaterally with EGM-2 and removing any medium on the apical surface. Monolayers of each cell type were also prepared as controls, using the same seeding densities and culture methods/feeding intervals for comparison.

### BCG growth and culture

An aliquot (50 μL) of frozen *M. bovis* BCG Pasteur glycerol stock (University of Surrey culture collection) was used to streak a previously prepared Middlebrook 7H11 agar plate (BD Biosciences, San Jose, CA). The plate was cultured for 7 days at 37 °C (ambient CO_2_) supplemented with Oleic Acid, Albumin Dextrose, Catalase (OADC) growth supplement (Sigma, St.Louis, MI). At this point, one colony was used to inoculate 5 mL Middlebrook 7H9 broth (BD Biosciences) supplemented with OADC; this was cultured at 37 °C, shaken at 200 rpm for 7 days. This culture was designated p1. Subculture was performed by taking 1 mL of p1 and using to inoculate 20 mL 7H9 broth (designated p2). Following a further culture period of 7 days as before, cultures were adjusted to an optical density at 600 nm of 0.5 (~ 10^7^ cfu/mL). Glycerol stocks were prepared by adding 500 μL bacterial culture to 500 μL 50% glycerol. Stocks were stored in cryovials at -80 °C. These stocks were used for all subsequent experimental infections.

### Infection of co-culture model with BCG

Prior to infection, all medium was removed, and co-cultures were gently washed once with Dulbecco’s Phosphate Buffered Saline (DPBS) (Thermo Fisher Scientific, Waltham, MA) as previously described^36^. EGM-2 (600 μL) was added to the basolateral chamber. Frozen samples of bacteria were thawed and spun in a microcentrifuge for 2 min at 13,000 × *g.* Each pellet was resuspended in 100 μL of EGM-2 and vortexed vigorously for 30 s. This suspension of ~ 10^7^ CFU was diluted to 1 mL EGM-2, adding 100 μL to the apical chamber of each insert to give an approximate multiplicity of infection of 10:1 (BCG to BATII). Plates were incubated at 37 °C in 5% CO_2_ for 4, 24, or 48 h. At each time point all contents of the basolateral chamber were collected and centrifuged at 13,000 × *g* for 2 min in a microcentrifuge to pellet the bacteria and cells. Pellets were resuspended in 0.1% Triton X-100/DPBS (Sigma) and vortexed vigorously for 30 s. Ten-fold serial dilutions were plated on Middlebrook 7H11 agar and cultured for 21–28 days. The number of CFU for each incubation time point were divided by the product of the dilution and the plated volume, averaged, and reported as cfu/mL. Numbers in the apical chamber were determined similarly. To determine numbers of cell and membrane associated bacteria, membranes were washed twice with phosphate-buffered saline before 0.5 mL of 0.1% Triton X-100/DPBS was added to each chamber. Membranes were then removed from the plastic supports, and minced well with a sharp scalpel. Membrane fragments suspended in 0.1% Triton X-100/DPBS were vortexed, and the suspension was diluted and plated as before.

### Immunohistochemistry and in situ visualisation of BCG

Whole inserts were fixed in neutral buffered formalin (Sigma) and were dehydrated sequentially in 70, 90 and 100% ethanol, for 30 min at each concentration. These were then incubated in 100% isopropanol for 30 min, transferred to molten paraffin wax (65 °C) for 1 h and the membrane excised from the insert using a scalpel blade. The membrane was then embedded in paraffin, cured and sectioned at 4 μm.

For H&E staining, sections were dewaxed and stained as outlined previously^80^. Stained sections were dehydrated and mounted in DPX mounting medium (Sigma) with a cover-slip overlaid for analysis on a Nikon Eclipse Ci upright microscope. To visualise BCG in the co-cultures, sections were dewaxed and stained according to the Ziehl–Neelsen protocol. Briefly, deparaffinised sections were placed into pre-heated ready-to-use Carbol-Fuchsin solution (Sigma) at 58 °C water bath for 15 min. Slides were then dipped into tap water for 2 min (to avoid loss of delicate sections). Sections were differentiated in 3% hydrochloric acid in 95% ethyl alcohol (both Sigma) and washed briefly before counterstaining 1.4% methylene blue (Sigma) for 15 s. Finally, slides were washed and mounted as per H&E sections.

### Evaluation of co-culture barrier integrity

Methods to evaluate co-culture barrier integrity have been previously described^36^. Briefly, to determine integrity of the co-culture, the extent of tight junction formation and the effect of BCG infection on integrity, growth medium was removed at the end of each experiment and inserts were transferred to a fresh plate containing 600 μL DPBS in each well. During transit, 100 μL of 10 mg/mL Blue Dextran 2000 kDa (DB2000) (Sigma) in DPBS was added to the apical surface. After 1 h, inserts were removed from wells and 40 uL of the apical solution removed. This was diluted 1/15 to bring samples within range of the standard curve. Optical densities were determined on an Eppendorf BioSpectrometer (Eppendorf AG, Hamburg, Germany), together with undiluted basolateral samples. Standards of Blue Dextran in DPBS were prepared at 5, 2.5, 1.25, 0.625, 0.3125, 0.156, 0.078 and 0.039 mg/mL. Controls consisted of a blank insert, no membrane control and monocultures of either BATII or BPAEC, to provide an indication of relative contribution to resistance.

To provide a real time indication of tight junction formation, trans-epithelial electrical resistance (TEER) was measured between the point of seeding until the day of harvest, as described previously^36^. Measurements were taken using an EVOM2 Voltohmmeter with STX-2 chopstick electrodes (World Precision Instruments, Stevenage, UK) immediately before the medium was exchanged. For measurements, 0.5 mL and 1.0 mL of medium were added to the apical and basolateral chambers, respectively, allowing the medium to equilibrate to 37 °C before measurements were performed in triplicate. All values were converted to Ohms/cm^2^ using Eq. (1)

1 $$ {\text{Final TEER }}\left( {\Omega /{\text{ cm}}^{{2}} } \right) \, = {\text{ Net TEER }}\left( \Omega \right) \, \times {\text{ Area of Transwell insert }}\left( {{\text{cm}}^{{2}} } \right) $$

### Immunofluorescence

To study the expression of MHC Class I, II and the accessory molecule CD54 in the presence of BCG infection, BATII cells were seeded onto Nunc Lab-Tek II 8 chamber coverglass slides (Thermo Fisher Scientific), as outlined previously^36^. Cells were seeded at 4 × 10^4^ cells per chamber and cultured for 48 h, before infection with BCG at a multiplicity of infection (MOI) of 10. Slides were returned to the incubator for 24 h before washing with DPBS and fixed in 4% PFA (Sigma) at room temperature for 15 min, before permeabilisation by washing in an immunofluorescence wash buffer (IF buffer, recipe as published previously^36^) containing 0.1% Triton X-100 for 15 min. Blocking was performed for 1 h at room temperature in DPBS/ 5% normal goat serum/ 0.1% Triton X-100 (Sigma). Primary antibodies (see below) were diluted 1/100 in blocking buffer, applied to cells and incubated overnight at 4 °C. Cells were rinsed three times with IF buffer and secondary antibody (see below) applied for 1 h at room temperature in the dark. Cells were again rinsed three times with IF buffer before adding Atto 565-labelled phalloidin (Sigma) according to manufacturer’s instructions (diluted in DPBS), along with 2 drops NucBlue DNA stain per mL (Thermo Fisher Scientific). This was removed and replaced with 200 uL DPBS for imaging on a Nikon Eclipse Ti confocal microscope, 40 × objective, using 488, 565 and 460 nm lasers.

For immunofluorescence (IF) analysis of co-cultures, inserts were washed with DPBS and processed as for coverglass monolayer cultures as per previously published protocols^36^. All primary antibodies were diluted 1/100 in blocking buffer, as before. Following the final wash to remove the secondary antibody, the membrane was removed from the insert using a scalpel blade, then mounted in Prolong Gold Antifade reagent with 4′,6-diamidino-2-phenylindole (DAPI) (Thermo Fisher Scientific) or Vectashield Hardset mounting medium with phalloidin (TRITC) (Vector Laboratories, Burlingame, CA) between two cover slips. Co-cultures were imaged using a Nikon Eclipse Ti confocal microscope (Nikon Corporation, Tokyo, Japan).

Primary antibodies are as follows: MHC Class I and II were generated in the laboratory of Bernardo Villarreal-Ramos and Martin Vordermeier (Animal and Plant Health Agency, Weybridge, UK). Pro-surfactant protein C (SFTPC) was sourced from Millipore (Product ab3786; Millipore, Burlington, MA) and CD31 (clone HEC7) from Fisher (Product MA3100; Thermo Fisher Scientific). Isotype controls were used accordingly (Sigma).

### LDH-Glo, Caspase-Glo 3/7 and CellTiter-Glo assays

All kits were sourced from Promega (Promega Corporation, Madison, WI) and used according to manufacturer’s instructions. To detect lactate dehydrogenase in the culture medium, 2 μL of medium was removed and diluted 50-fold into storage buffer. LDH detection reagent was added at a 1:1 ratio and the plate incubated at room temperature for 60 min, before reading luminescence using the CLARIOstar plate reader (BMG LABTECH, Aylesbury, UK). Both Caspase-Glo substrate and CellTiter-Glo reagent were added directly to the culture plate at a 1:1 ratio with medium and incubated for 10 min (CellTiter-Glo) or 30 min (Caspase-Glo) at room temperature before reading on the CLARIOstar plate reader.

### Cytokine quantification by ELISA

Culture supernatants were collected in triplicate and kept at  − 20 °C until use. ELISAs were performed for the cytokines IL8 (Kingfisher Biotech, St.  Paul, MN), TNFα (Kingfisher Biotech), IL17a (Kingfisher Biotech) and IL22 (CUSABIO, Houston, TX) as described by the manufacturer. Briefly, for Kingfisher sourced kits (IL8, TNFα, IL17a), manufacturer supplied standard and samples were added to a 96-well plate and incubated at room temperature for 1 h, washed and incubated with detection antibody for 1 h at room temperature. The plate was washed and streptavidin-HRP working solution was added and the plate incubated for 30 min at room temperature. Following a further wash, 3,3′,5,5′-tetramethylbenzidine (TMB) substrate was added and the plate developed in the dark for 30 min at room temperature. Optical density was read at 450 nm with a CLARIOstar plate reader. Sample concentrations were interpolated from the standard curve. The CUSABIO sourced IL22 ELISA differed in that standards and sample were incubated at 37 °C for 2 h, detection antibody for 1 h at 37 °C, streptavidin-HRP working solution for 1 h at 37 °C, and TMB substrate for 30 min at 37 °C.

### Infection of PBMCs with *M. bovis* BCG

Cryovials containing frozen PBMCs from vaccinated (1412) and naïve (1406) cattle, received from the laboratory of Martin Vordermeier and Bernardo Villareal-Ramos at the Animal and Plant Health Agency were removed from liquid nitrogen and transferred to a 37 °C water bath. The cells were thawed quickly by holding the cryovials in the water bath occasionally tapping the cryovial. Thawed cells were transferred into a 15 mL conical tube containing 10 mL room temperature OpTmizer CTS serum free medium (Thermo Fisher Scientific). Cells were pelleted at 300 × *g* for 5 min at room temperature before performing a Trypan Blue viability assay using the TC-20 Automated Cell Counter as before. The final concentrations of the cells were adjusted to 2 × 10^6^/mL at room temperature in OpTmizer CTS, seeding 0.5 mL (1 × 10^6^) per well in a 24 well plate format. Each well was infected with 0.5 mL of *M. bovis* BCG at 2 × 10^6^/mL (final seeding density 1 × 10^6^ per well, MOI of 1). Plates were incubated at 37 °C, 5% CO_2_, 95% air for 7 days. Uninfected wells had 0.5 mL OpTmizer CTS medium added. Supernatant was harvested from all wells, pooled according to infection status and filtered to 0.2 μm, before storing at − 20 °C until ready for use. Supernatant at this stage was considered Conditioned Medium (CM).

### Addition of CM to co-cultures and infection with *M. bovis* BCG

Co-cultures were generated using 6.5 mm diameter (0.33 cm^2^) Transwell-CLEAR 8 μm pore size permeable membranes in a 24-well plate as before, culturing at ALI for 14 days before treatment with CM derived from infected (+) or uninfected (−) PBMC cultures (see Table 1 for full details of treatment groups). On the day of treatment, EGM-2 was removed from the basolateral compartment and any residual medium aspirated gently from the apical surface of the culture. CM (500 μL) was added to the basolateral chamber of each insert, each group containing 6 replicates (half of which would be later infected with BCG). OpTmizer CTS medium was added to three co-cultures to act as a vehicle only control, whilst three further inserts were treated with their usual EGM-2 medium to act as untreated controls. Co-cultures were returned to the incubator (37 °C, 5% CO_2_, 95% air) for 24 h, before infection with BCG in triplicate at an MOI of 10 (to the seeding density of BATII cells; see ‘Infection of co-culture model with BCG’) or EGM-2 medium only as control. Medium was harvested for BCG quantification and cytokine analysis as outlined above.

### Statistical analysis

Analyses were performed using GraphPad Prism version 8.02 for Windows, GraphPad Software, La Jolla, CA, https://www.graphpad.com. Replicate numbers of samples and experiments can be found as part of figure legends. Blue Dextran measurements were analysed using a one-way ANOVA with a Tukey’s multiple comparisons test as follow on analysis, presenting values as Means ± Standard Deviation (SD), where n represents individual inserts. Statistical significance has been denoted as *p ≤ 0.05, **p ≤ 0.01, ***p ≤ 0.001 and ****p ≤ 0.0001 (Tukey’s multiple comparisons test only shown).

For TEER analysis, an average of three readings per insert was performed at each time point, with n = 3 inserts per group, per experiment. Data represents three experiments performed on separate occasions and presented as Means ± SD, analysed using an unpaired t-test.

## Supplementary information

Supplementary Information

## Data Availability

All data are available in their raw formats and without reservations upon request.
