# Supplementary material for: Modelling early events in Mycobacterium bovis infection using a co-culture model of the bovine alveolus
Source: Sci Rep. 2020 Oct 28;10:18495. doi: 10.1038/s41598-020-75113-6 (PMC7595104; doi:10.1038/s41598-020-75113-6)
Supplement: Supplementary file 1 — Supplementary Information [file 41598_2020_75113_MOESM1_ESM.pdf]

Modelling early events in Mycobacterium bovis infection using a co-culture model of the bovine alveolus

Diane Frances Lee*[1], Graham Roger Stewart [2] and Mark Andrew Chambers[1,2]

[1]School of Veterinary Medicine, University of Surrey, Guildford, Surrey, UK

[2]School of Biosciences and Medicine, University of Surrey, Guildford, Surrey, UK

*diane.lee@surrey.ac.uk

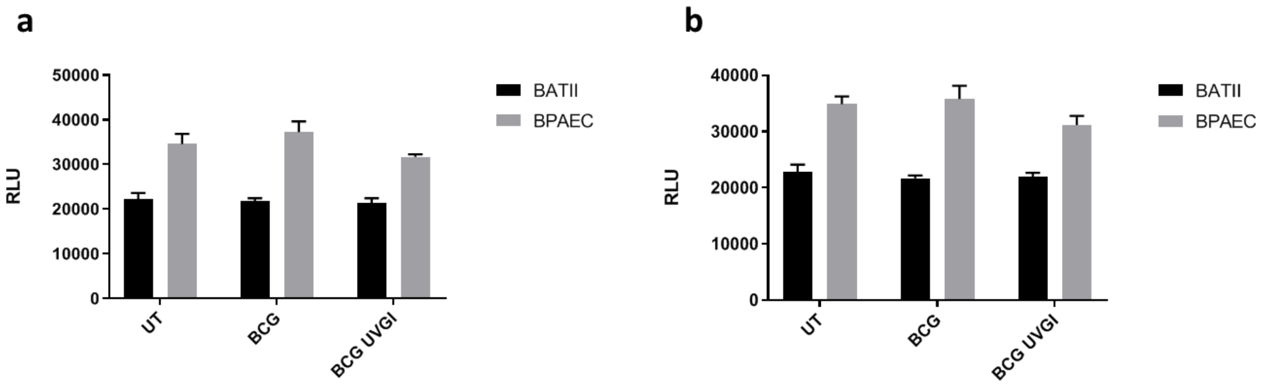

Supplementary Figure S1. Caspase 3/7 levels measured at a) 4 h and b) 24 h, showing no significant reductions in viability when infected with live BCG (Two-Way ANOVA, followed by Tukey's Multiple Comparisons Test; P ≥ 0.05. Data presented as Mean ± SD, n=3.

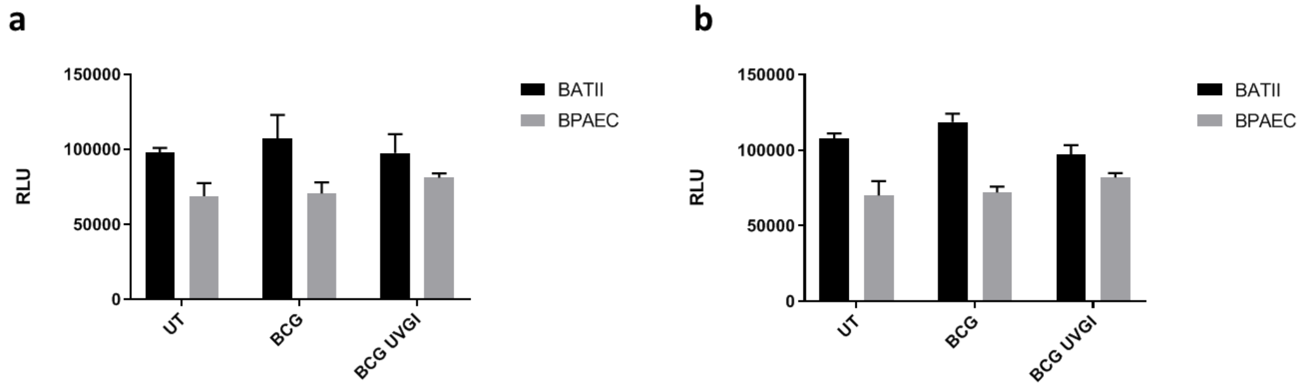

Supplementary Figure S2. Lactate Dehyrogenase (LDH) levels measured at a) 4 h and b) 24 h, showing no significant reductions in viability when infected with live BCG (Two-Way ANOVA, followed by Tukey's Multiple Comparisons Test; P ≥ 0.05. Data presented as Mean ± SD, n=3.

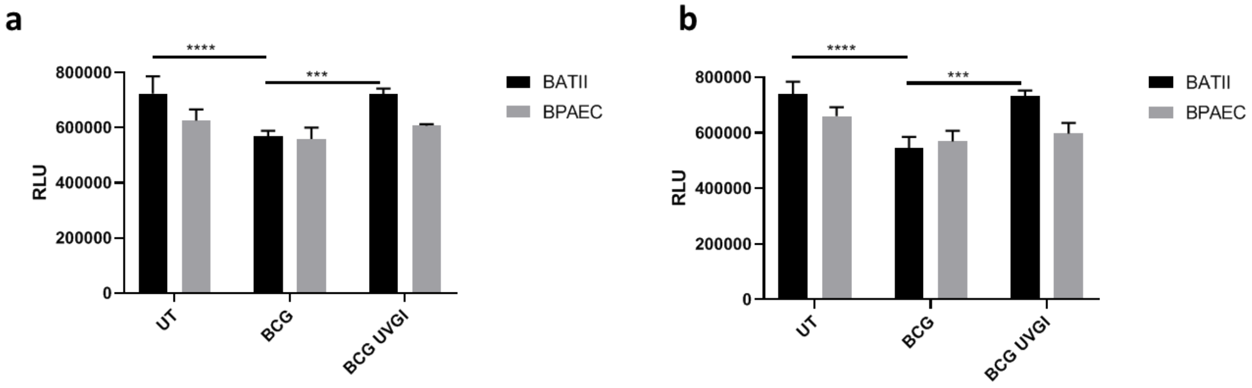

Supplementary Figure S3. CellTiter-Glo at a) 4 h and b) 24 h, showing significant reductions in viability when infected with live BCG (Two-Way ANOVA, followed by Tukey's Multiple Comparisons Test). Data presented as Mean ± SD, n=3; ***P ≤ 0.001, ****P ≤ 0.0001.
